# Supplementary material for: Myoferlin Is a Yet Unknown Interactor of the Mitochondrial Dynamics’ Machinery in Pancreas Cancer Cells
Source: Cancers (Basel). 2020 Jun 21;12(6):1643. doi: 10.3390/cancers12061643 (PMC7352660; doi:10.3390/cancers12061643)
Supplement: Supplementary file 1 [file cancers-12-01643-s001.zip › cancers-824133-suppl-final/cancers-824133-supplementary.pdf]

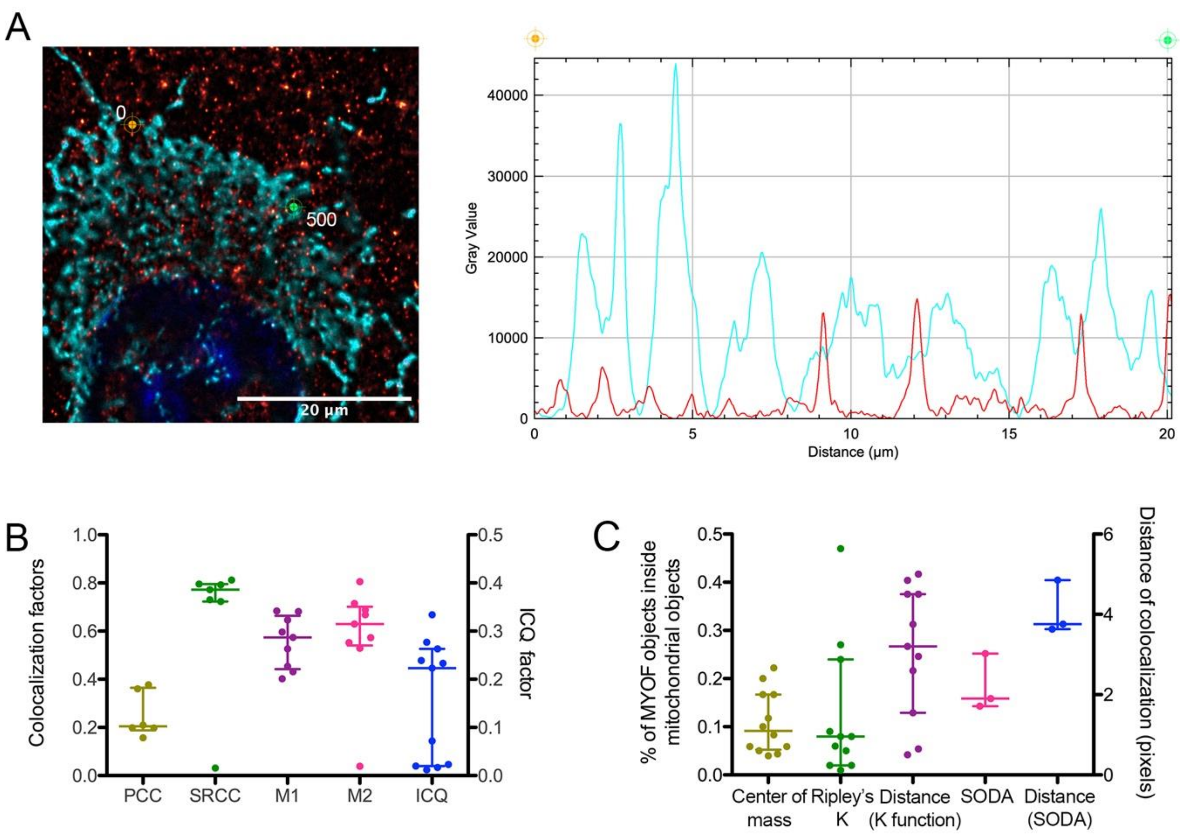

Figure S1. Myoferlin was colocalized with mitochondria in Panc-1 cells. (**A**) Deconvoluted confocal image of nuclei (blue), myoferlin (HPA - "hot" red scale), mitochondria (113-1 - "cold" cyan scale). Scale bar = 20 µm. Channel intensity profile was established following the segment between orange (0-pixel position) and green (500-pixel position) cross marks. (**B**) Pearson (PCC), Spearman rank (SRCC) correlation coefficients, Manders' colocalization coefficients (M1, M2), and intensity correlation quotient (ICQ) calculated on 11 independent microscopic fields. (**C**) Percentage of myoferlin-positive objects (N=7365) with center of mass overlapping mitochondrial object (N=273), percentage of myoferlin-positive object colocalizing mitochondrial object calculated by fitting of the Ripley's K function or by statistical object distance analysis (SODA). Colocalization distances in pixels were measured in both cases.

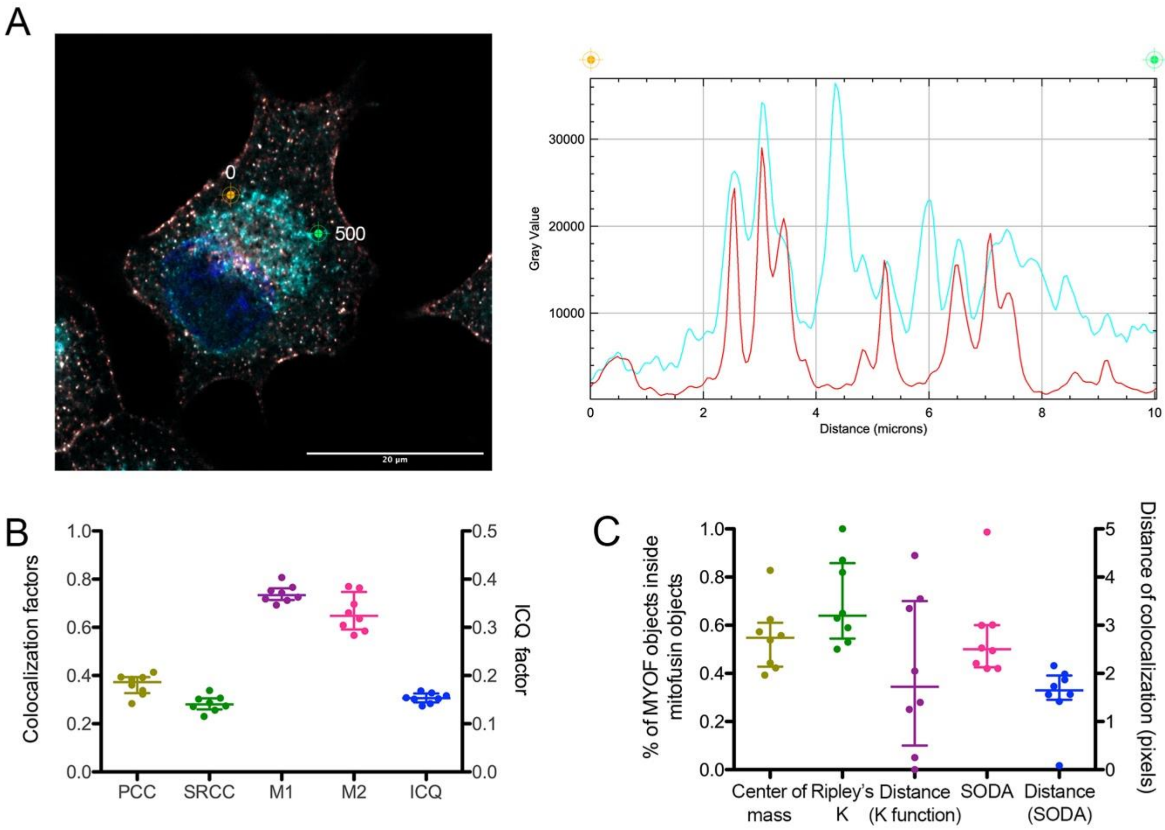

Figure S2. Myoferlin was colocalized with mitofusins in Panc-1 cells. (A) Deconvoluted confocal image of nuclei (blue), myoferlin (HPA - "hot" red scale), mitofusin-1/2 (3C9 - "cold" cyan scale). Scale bar = 20 µm. Channel intensity profile was established following the segment between orange (0-pixel position) and green (500-pixel position) cross marks. (B) Pearson (PCC), Spearman rank (SRCC) correlation coefficients, Manders' colocalization coefficients (M1, M2), and intensity correlation quotient (ICQ) calculated on 8 independent microscopic fields. (C) Percentage of myoferlin-positive objects (N=7128) with center of mass overlapping mitofusin-positive object (N=369), percentage of myoferlin-positive object colocalizing mitofusin-positive object calculated by fitting of the Ripley's K function or by statistical object distance analysis (SODA). Colocalization distances in pixels were measured in both cases.

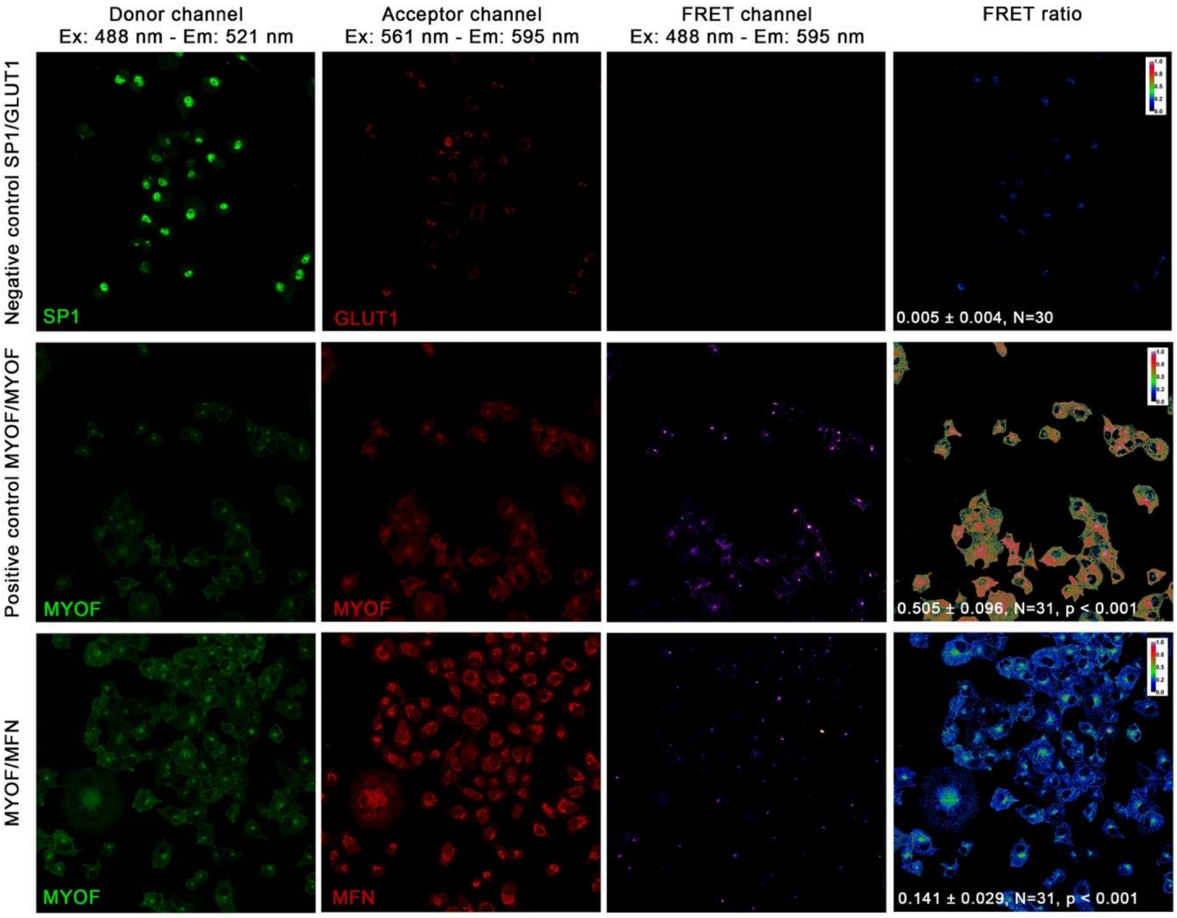

Figure S3. Fluorescence resonance energy transfer showing myoferlin and mitofusins proximity in Panc-1 cell line. Confocal images of myoferlin (HPA - green) and mitofusin-1/2 (3C9 - red) immunofluorescence. Fluorescence resonance energy transfer (FRET) channel was represented with "fire" color scale. FRET ratio was represented with a "rainbow" color scale associated with mean ± standard deviation.

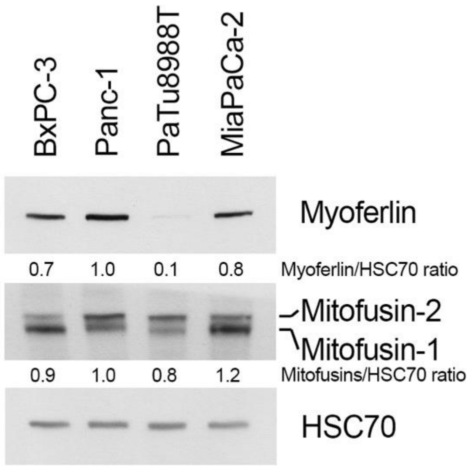

Figure S4. Myoferlin and mitofusins abundance in PDAC cell lines. Western-blot of 20 μg protein samples from PDAC cell lines. Myoferlin and mitofusins were detected on the same membrane. HSC70 was used as a loading control. Relative quantification was performed using ImageJ.
