# Supplementary figures and images for: Myoferlin Is a Yet Unknown Interactor of the Mitochondrial Dynamics’ Machinery in Pancreas Cancer Cells
Source: Cancers (Basel). 2020 Jun 21;12(6):1643. doi: 10.3390/cancers12061643 (PMC7352660; doi:10.3390/cancers12061643)

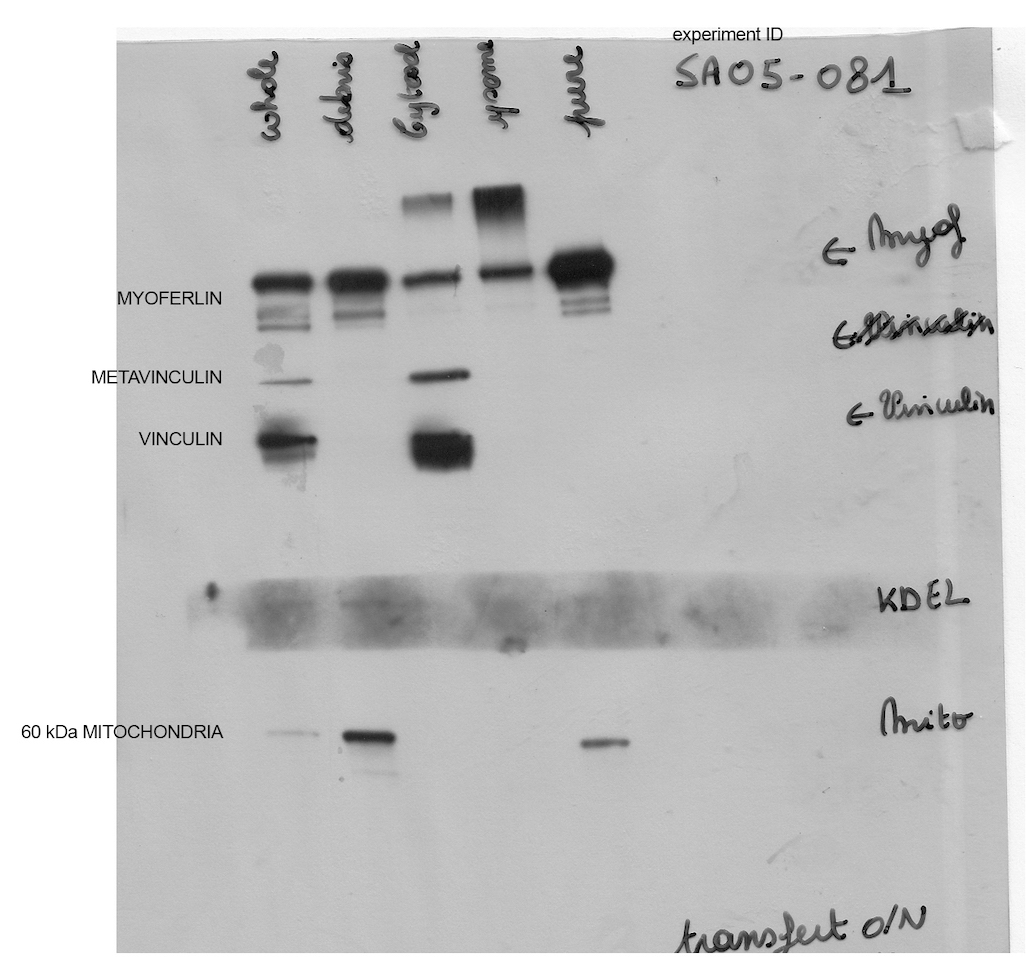

Supplement: Supplementary file 1 [file cancers-12-01643-s001.zip › cancers-824133-suppl-final/Full WB/F1A full.jpg]

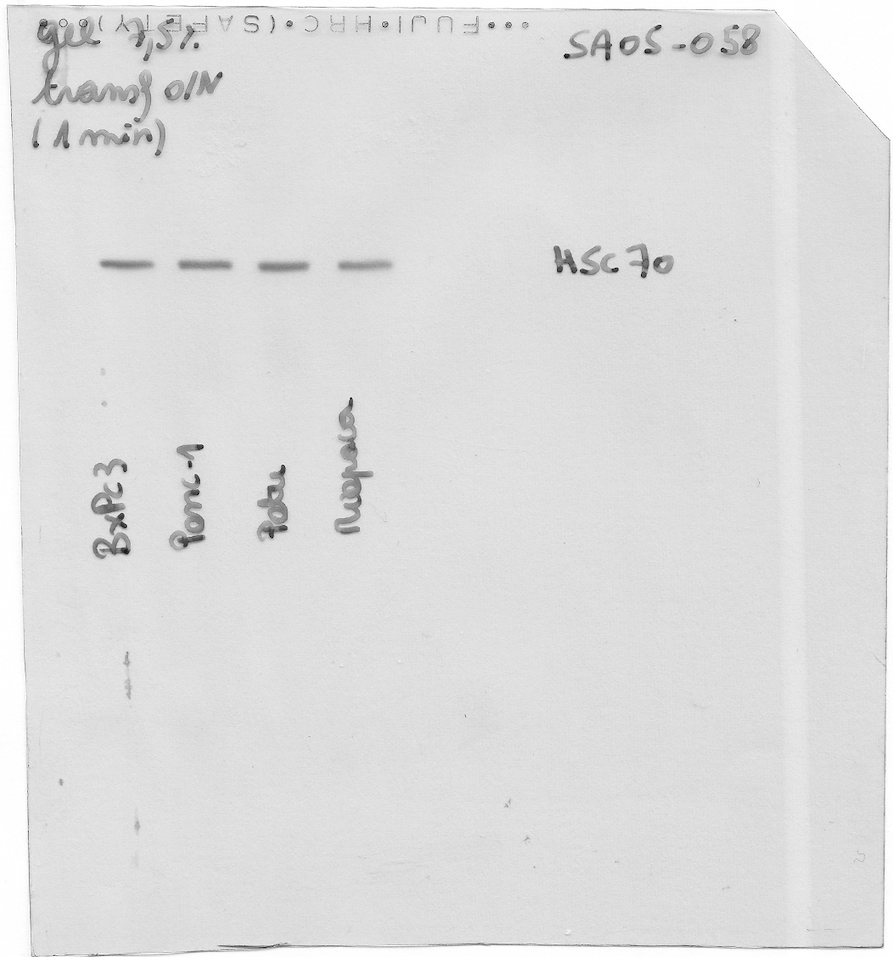

Supplement: Supplementary file 1 [file cancers-12-01643-s001.zip › cancers-824133-suppl-final/Full WB/F3S full hsc70.jpg]

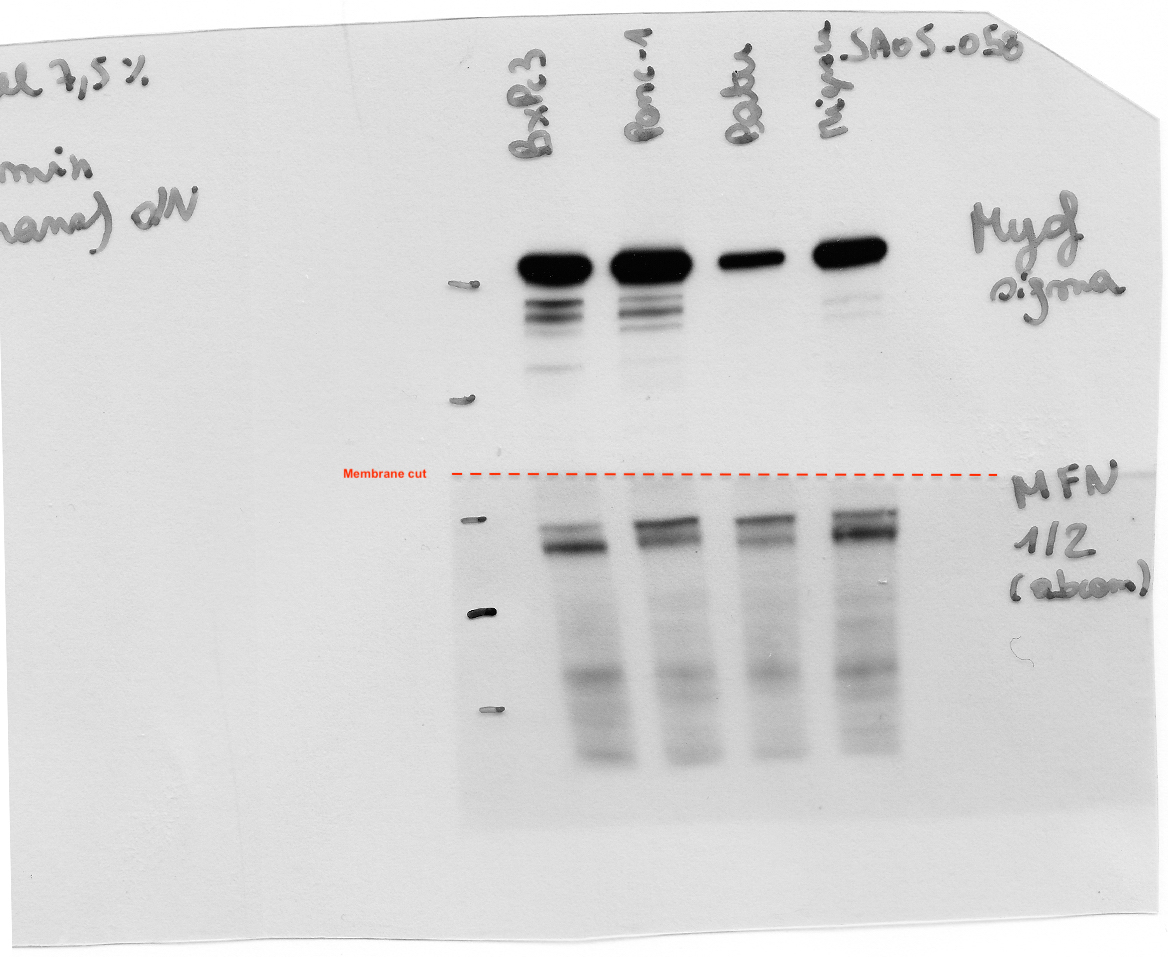

Supplement: Supplementary file 1 [file cancers-12-01643-s001.zip › cancers-824133-suppl-final/Full WB/F3S full mfn.jpg]

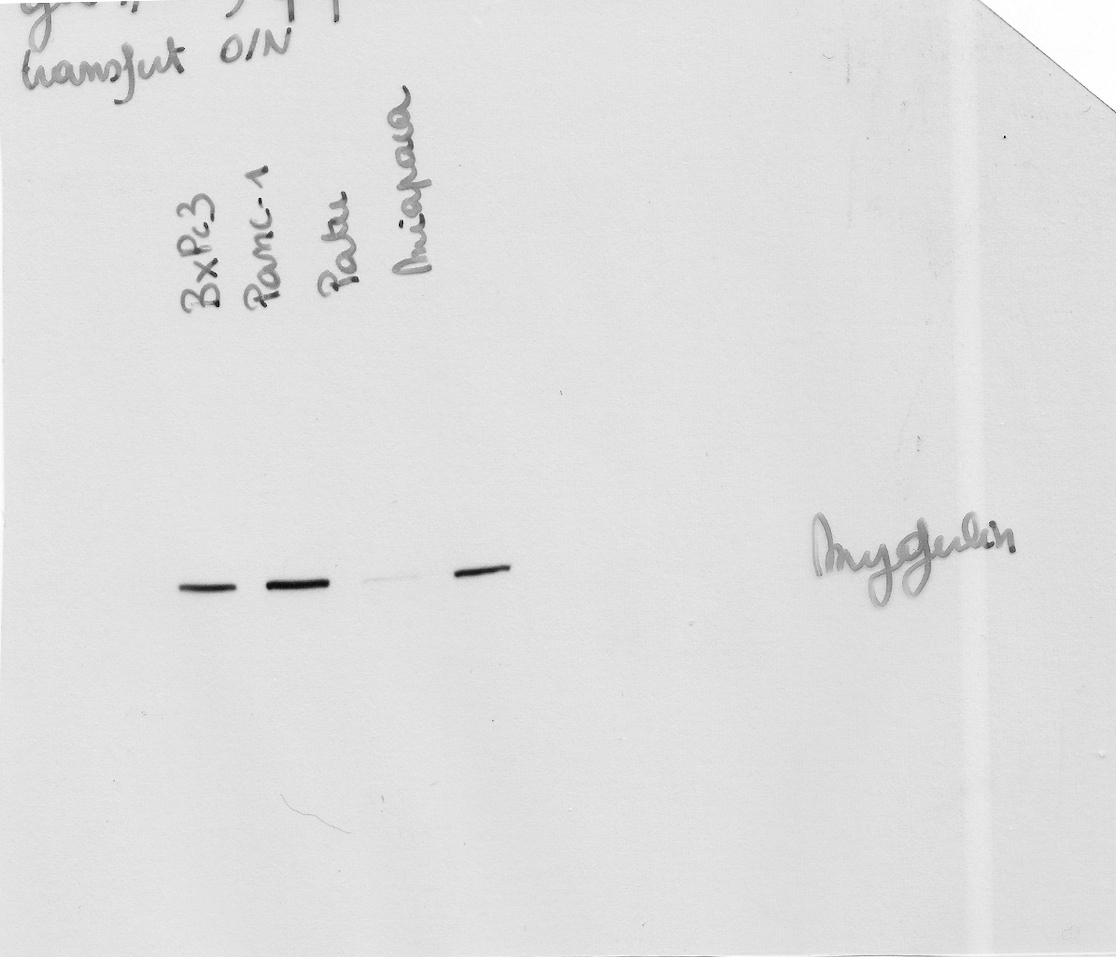

Supplement: Supplementary file 1 [file cancers-12-01643-s001.zip › cancers-824133-suppl-final/Full WB/F3S full myoferlin.jpg]

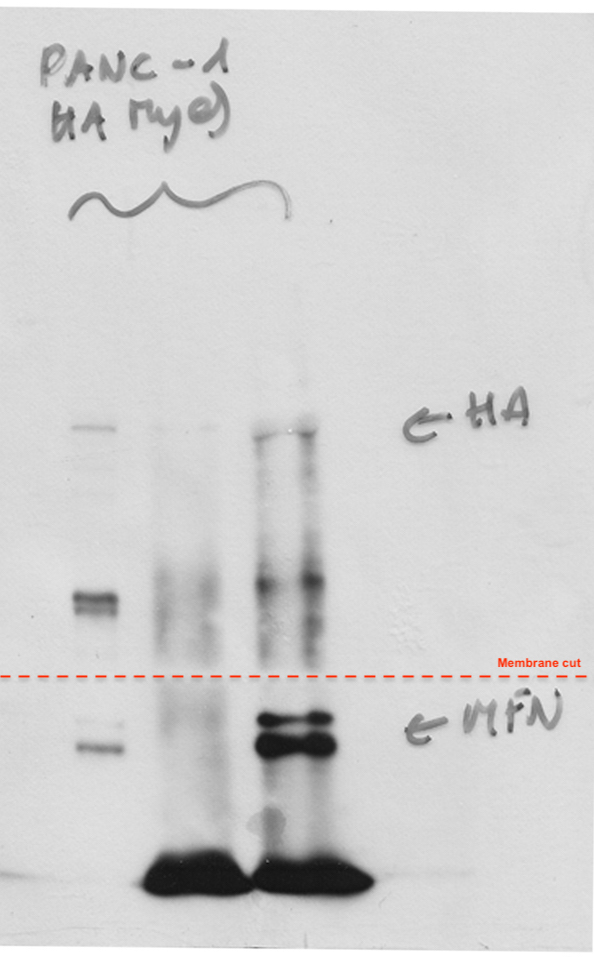

Supplement: Supplementary file 1 [file cancers-12-01643-s001.zip › cancers-824133-suppl-final/Full WB/F4A full.jpg]

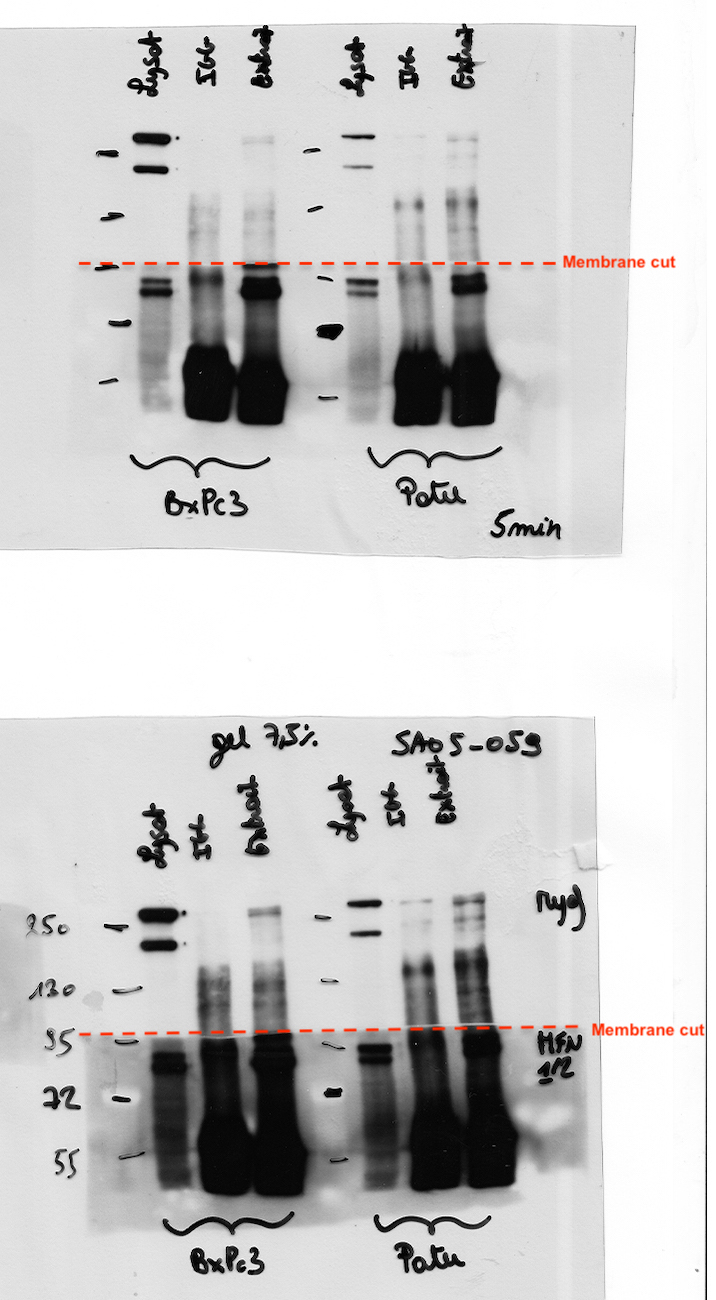

Supplement: Supplementary file 1 [file cancers-12-01643-s001.zip › cancers-824133-suppl-final/Full WB/F4B full BX-Patu full.jpg]

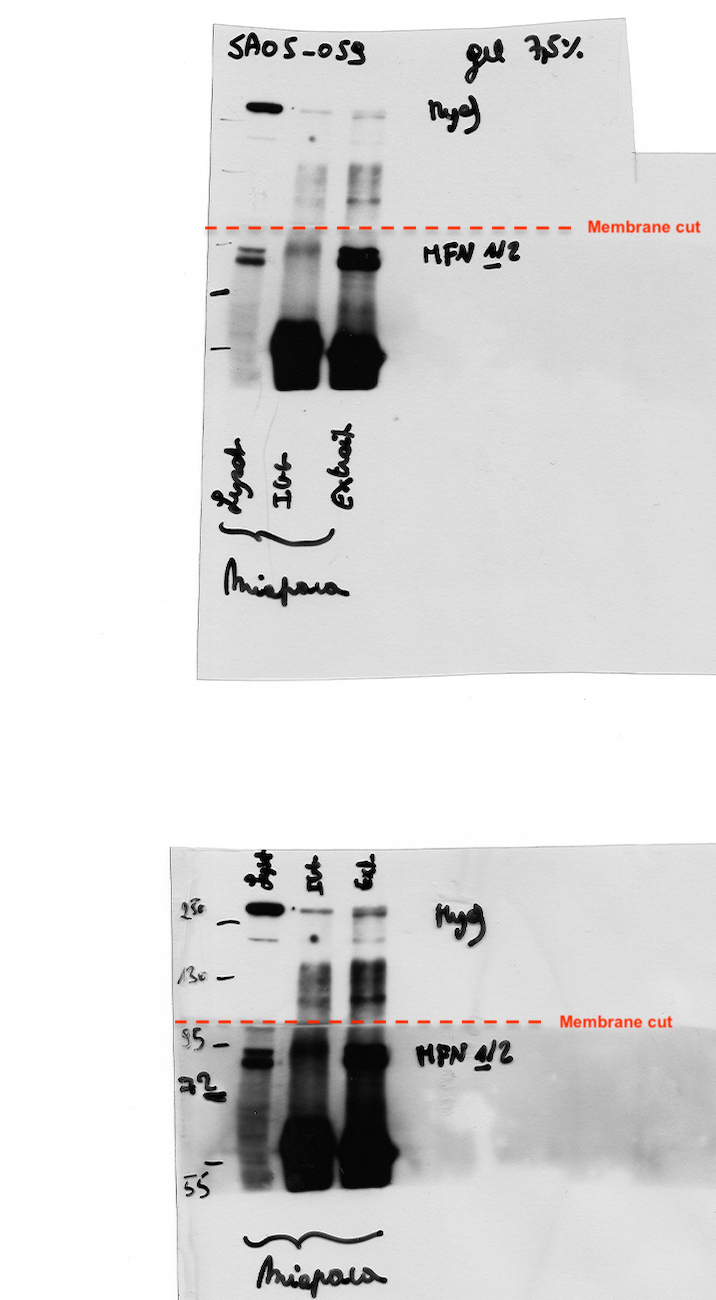

Supplement: Supplementary file 1 [file cancers-12-01643-s001.zip › cancers-824133-suppl-final/Full WB/F4B full miapaca full.jpg]

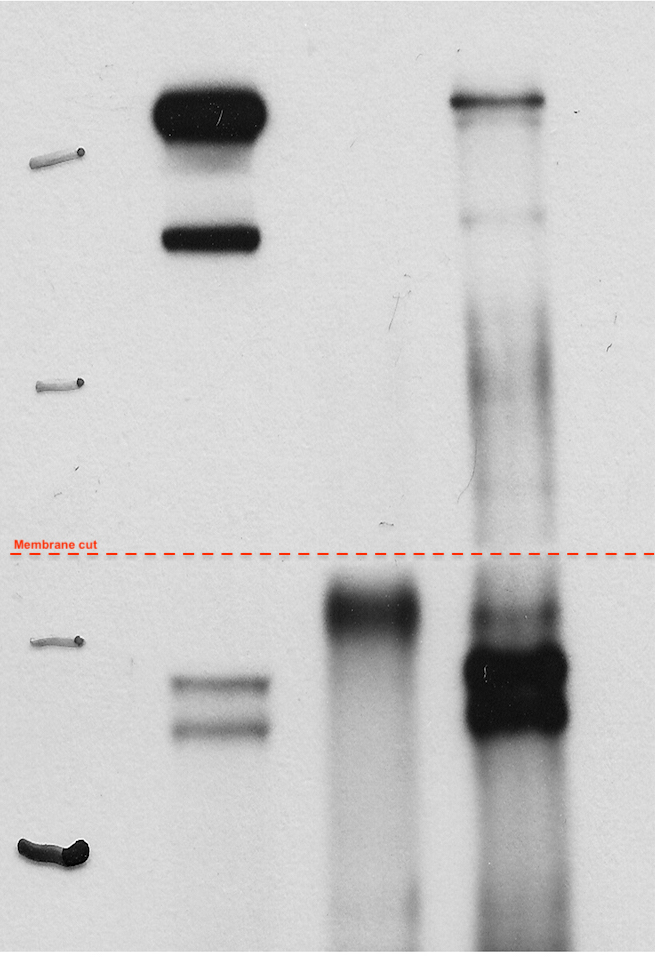

Supplement: Supplementary file 1 [file cancers-12-01643-s001.zip › cancers-824133-suppl-final/Full WB/F4B full PANC1.jpg]

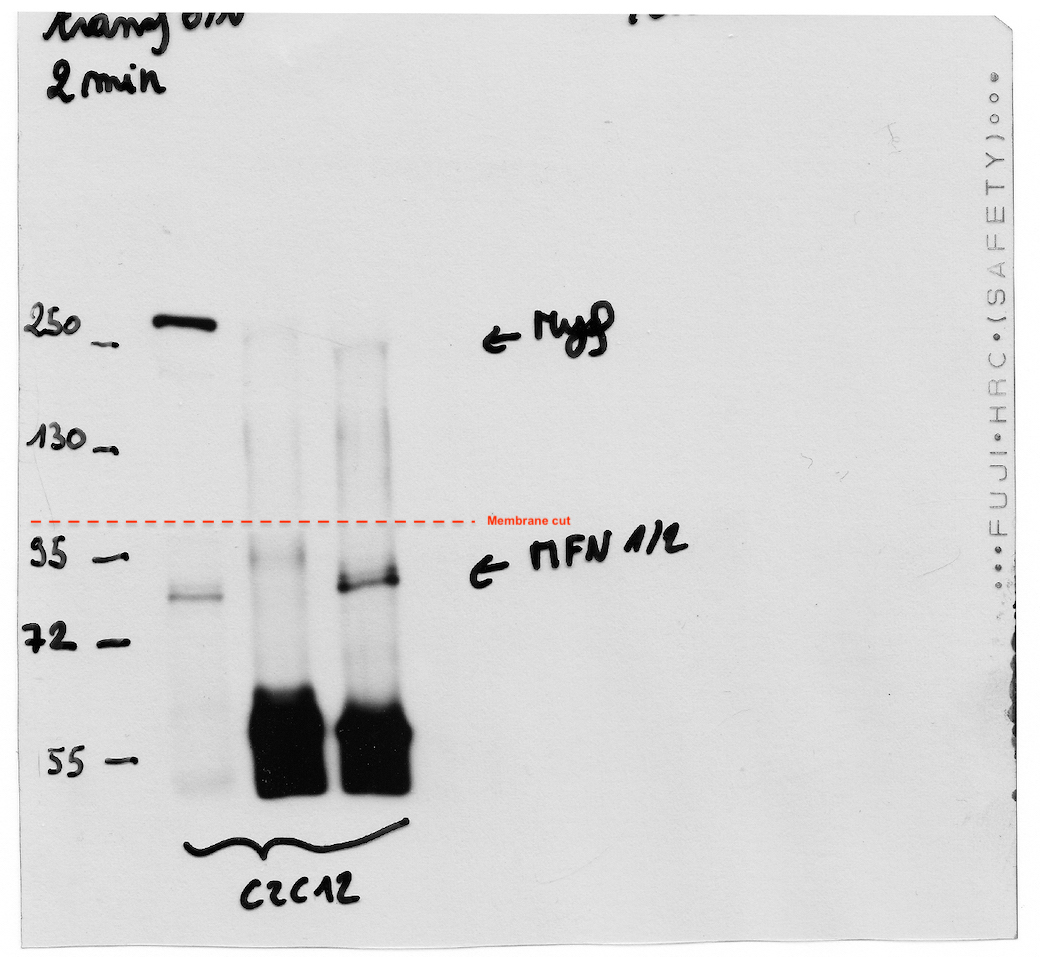

Supplement: Supplementary file 1 [file cancers-12-01643-s001.zip › cancers-824133-suppl-final/Full WB/F6 full C2C12.jpg]

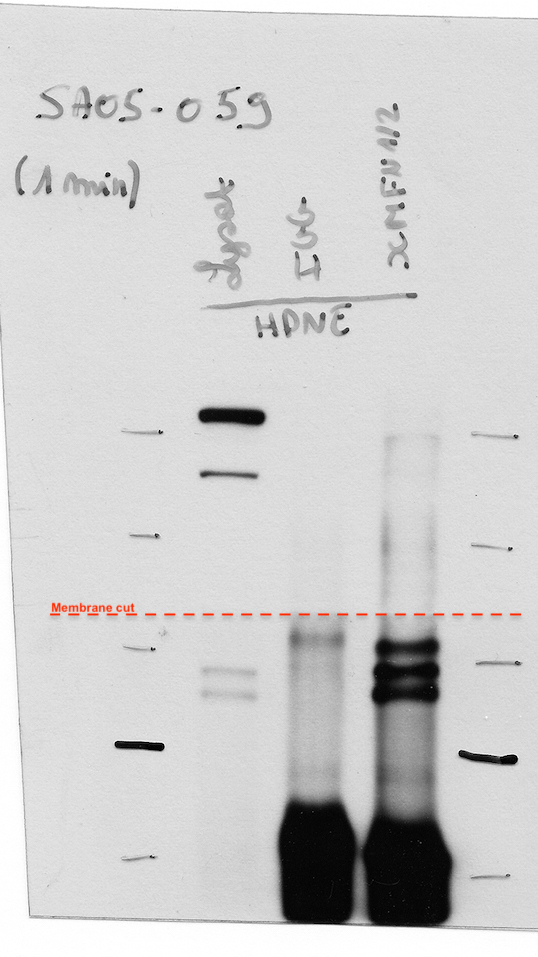

Supplement: Supplementary file 1 [file cancers-12-01643-s001.zip › cancers-824133-suppl-final/Full WB/F6 full HPNE.jpg]
